# A rare case of cavernous hemangioma accompanied with diffuse hepatic hemangiomatosis

**DOI:** 10.1186/s40792-020-01023-4

**Published:** 2020-10-01

**Authors:** Takuji Ota, Toshiya Kamiyama, Takuya Kato, Takayuki Hanamoto, Kunihiro Hirose, Noriyuki Otsuka, Shinichi Matsuoka, Akinobu Taketomi

**Affiliations:** 1 Surgery Department, Tomakomai General Hospital, 1-5-20 Shimizu-cho, Tomakomai, Hokkaido Japan; 2 Department of Gastroenterological Surgery I, Hokkaido University Graduate School of Medicine, Kita 14, Nishi 5, Kita-ku, Sapporo, Hokkaido Japan; 3 Department of Pathology, Graduate School of Medicine/Faculty of Medicine, Hokkaido University, Kita 14, Nishi 5, Kita-ku, Sapporo, Hokkaido Japan

**Keywords:** Hemangiomatosis, Hemangioma, Oral contraceptives

## Abstract

**Background:**

Hepatic cavernous hemangioma (CH) is the most common hepatic benign tumor. Most cases are solitary, asymptomatic, and found incidentally. In symptomatic cases with rapidly growing tumors and coagulopathy, surgical treatment is considered. In rare cases, diffuse hepatic hemangiomatosis (DHH) is reported as a comorbidity. The etiology of DHH is unknown.

**Case presentation:**

A 29-year-old female patient had a history of endometriosis treated with oral contraceptives. Hepatic CH was incidentally detected in the segment IVa of the liver according to the Couinaud classification. Follow-up computed tomography (CT) and ultrasound sonography showed the growth of the lesion and formation of multiple new lesions near the first. Enhanced CT and magnetic resonance imaging (MRI) revealed that the new lesions were different from CH. Although oral contraceptives were stopped, all lesions grew in size. Malignancy and possibility of rupture of these tumors were considered due to the clinical course, and we opted for surgical removal of the tumors. Left liver lobectomy and cholecystectomy were performed. Surgical findings were small red spot spreading and a mass in segment IV of the liver. Pathological examination revealed a circumscribed sponge-like tumor with diffuse irregular extension to the adjacent area. Both of the lesions consisted of blood-filled dilated vascular spaces lined by flat endothelium without atypia. The diagnosis was hepatic CH with DHH. The patient was discharged on postoperative day 12 uneventfully.

**Conclusion:**

We report the successful resection of CH with DHH. The case findings suggest a relationship between oral contraceptive use and enlargement of CH and DHH. Although DHH has been poorly understood, a few previously published cases reported DHH occurrence in patients using oral contraceptives. In such cases, the decision to perform surgical resection should be made after careful examination.

## Introduction

Hepatic cavernous hemangioma (CH) is the most common benign hepatic tumor. Most cases are solitary, asymptomatic, and found incidentally. Diffuse hepatic hemangiomatosis (DHH) is the replacement of the hepatic parenchyma with hemangiomatous lesions, and is associated with hereditary systemic diseases such as skeletal hemangiomatosis and Rendu–Osler–Weber disease. DHH without extrahepatic lesions is a rare condition in adults. The etiology of DHH is unknown, but it has been associated with the use of oral contraceptives. We present the case of a patient who was diagnosed with hepatic CH with DHH.

## Case presentation

A 29-year-old female was incidentally diagnosed with hepatic CH in liver segment IVb on abdominal CT and ultrasonography at the age of 25. She had a history of endometriosis treated with oral contraceptives starting at age 25. Follow-up imaging revealed that the lesion was growing and new, smaller lesions were distributed around the initial lesion. The oral contraceptives were immediately discontinued. The patient reported minimal alcohol consumption and no smoking history. The patient was 165.5 cm tall, weighed 87.5 kg (BMI = 31.9), and had no abnormalities of the skin, or laboratory values of blood counts, biochemistry, or coagulation tests. Hepatitis B and C virus markers were negative.

**Fig. 1:**
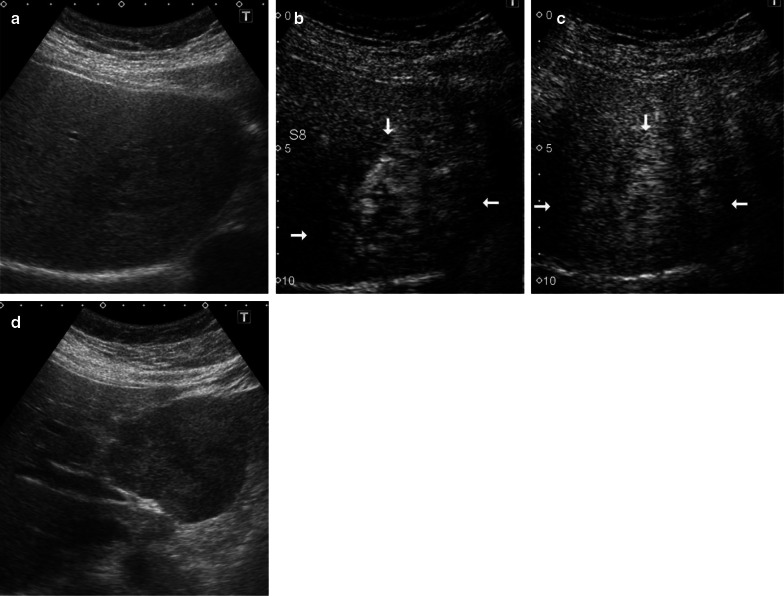
Sonographic findings. **a**–**c** Brightness mode and contrast-enhanced sonography reveal liner structures (white arrow) in liver segment IVa. **d** A 78-mm, hyperechoic mass in liver segment IVb has a hypoechoic mixed central region

**Fig. 2:**
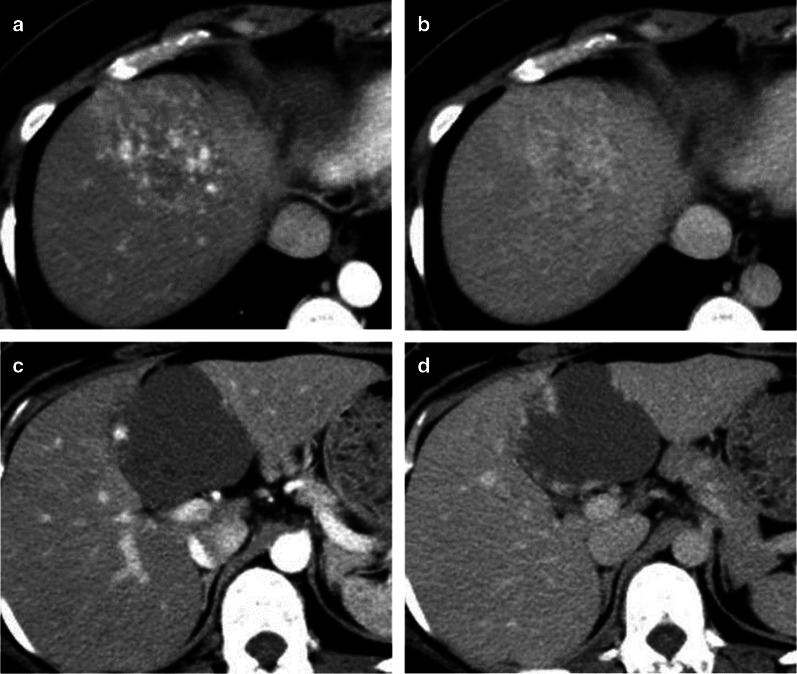
Enhanced CT findings. **a**, **b** Multiple ill-defined spots are heterogeneously enhanced in liver segment IVa. **c**, **d** An enhanced abdominal CT scan shows peripheral enhancement in the arterial phase (**c**) and fill-in contrast in the late phase (**d**) in liver segment IVb

**Fig. 3:**
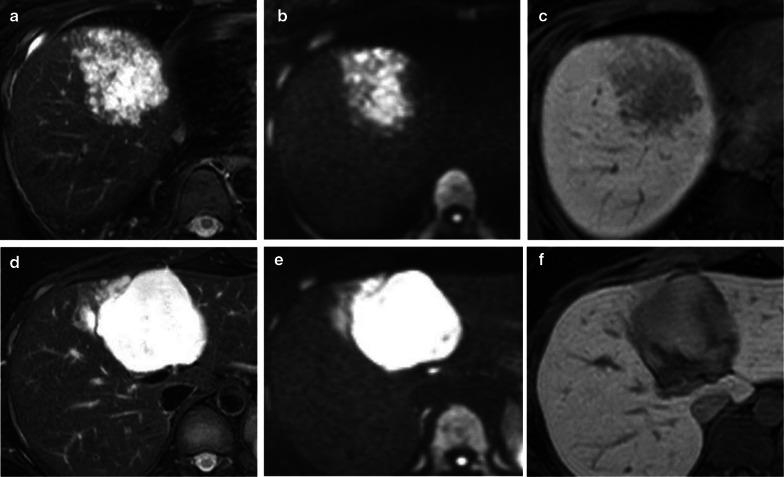
MRI findings. **a**–**c** In liver segment IVa, a T2-weighted image shows scattered high signal intensity (**a**). The lesions also show high signal intensity on diffusion-weighted images (**b**) and in the hepatobiliary phase of Gd-EOB-DTPA (**c**). **d**–**f** In liver segment IVb, T2-weighted and diffusion-weighted images show well-defined lesion with high signal intensity (**d**, **e**). The lesion is iso signal intensity in the hepatobiliary phase of Gd-EOB-DTPA (**f**)

An abdominal ultrasound (Fig. 1a–d) revealed a 78-mm marginal hyperechoic mass in liver segment IVb with a mixture of high and low echoes inside, and a contrast effect of a linear structure in liver segment IVa. CT imaging confirmed a 70-mm tumor in liver segment IVa (Fig. 2c, d) with peripheral enhancement that disappeared toward the center of the lesion. Dot-like nodules surrounded this lesion (Fig. 2a, b). The nodule in liver segment IVa had high signal intensity on T2-weighted images and diffusion-weighted images, with surrounding, smaller lesions. The lesions showed heterogeneous low signal intensity in the hepatobiliary phase of gadolinium-ethoxybenzyl-diethylenetriamine pentaacetic acid (Gd-EOB-DTPA) enhanced images (Fig. 3a–c). The well-defined tumor in segment IVb had high signal intensity on T2-weighted and diffusion-weighted images and in the hepatobiliary phase of Gd-EOB-DTPA enhanced images (Fig. 3d–f).

**Fig. 4:**
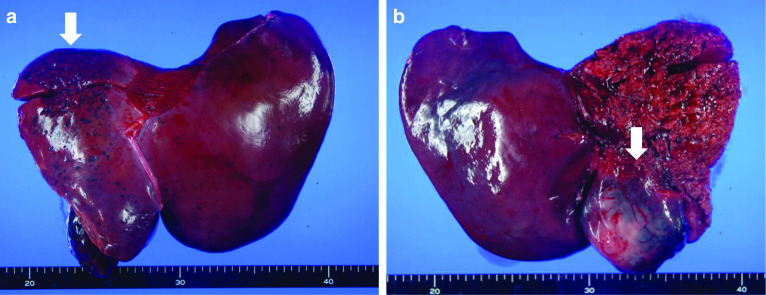
Resected specimen of left lobe of the liver. **a** Multiple lesions with small areas of small red patches (white arrow). **b** A well-defined lesion containing a 6.5-cm-sized blood clot (white arrow)

**Fig. 5:**
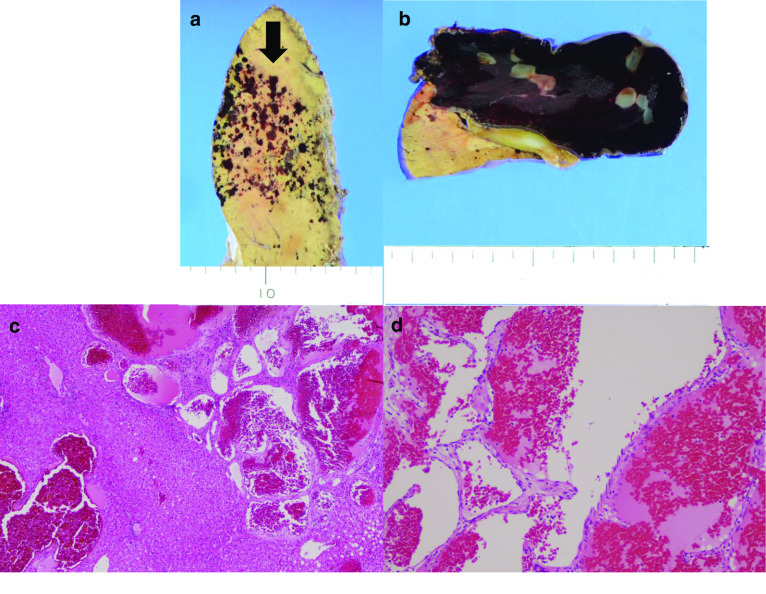
Histopathological examination of lesions in liver segment IV. **a**, **c** The disseminated red spots (black arrow) are areas in which blood vessels aggregated in liver segment IVa. These findings correspond with hemangiomatosis. **b**, **d** Microscopic findings of the well-defined tumor in liver segment IVb reveal that the vascular space is filled with blood and lined by endothelial cells, a common finding in CH

Due to the suspicion for malignancy and the risk of tumor rupture, a left liver lobectomy and cholecystectomy were performed. Macroscopic findings included the lesion in liver segment IVa with a small, surrounding area of small, red patches (Fig. 4a) and a well-defined lesion containing a 6.5-cm blood clot in liver segment IVb (Fig. 4b). Histopathologically, the irregularly scattered red spots were identified as aggregated blood vessels lined by endothelium without atypia. The distribution was disseminated, and the lesions were diagnosed as hemangiomatosis (Fig. 5a, c). The vascular spaces of the well-defined tumor in liver segment IVb were filled with blood and lined by flat endothelial cells. These are common findings for hepatic CH (Fig. 5b, d).

The patient’s postoperative course was uneventful and she was discharged on postoperative day 12. No recurrent liver lesions occurred as of 1 year after the operation.

## Discussion

Hepatic CH accounts for approximately 70% of benign liver tumors and is often found in women. Most cases involve single tumors, but multiple tumors are reported in 10% of cases [1]. The etiology of hepatic CH remains unknown. Pathologically, it has been found to consist of abnormal vascular tissue rather than neoplastic growth. Histologically, the spongy structure lined the vascular endothelium is filled with blood clots. Hepatic CH is generally asymptomatic, but treatment is required for tumors 10 cm or greater with a risk of rupture or when symptoms occur, such as abdominal symptoms including abdominal pain, or blood coagulation disorders, such as Kasabach–Merritt syndrome (KMS) [2, 3].

Hemangiomatosis is a vascular abnormality associated with hereditary systemic diseases, such as skeletal hemangiomatosis and Rendu–Osler–Weber disease. It is rare for hemangiomatosis to be restricted to the liver [4]. There were no findings suggestive of systemic hemangiomatosis in our patient. The CT revealed a clearly demarcated lesion, with a patchy inside and edges with gradually increased enhancement. In contrast, DHH is a nodular lesion with indistinct borders, and has been reported to be disseminated or to form a mass [5]. In our patient, typical findings of CH were observed in the contrast-enhanced mass in liver segment IV and we did not diagnose DHH preoperatively.

Our patient started taking oral contraceptives for endometriosis at the same time as the discovery of the hepatic CH. The rapid growth of new lesions in the liver during follow-up imaging resulted in the immediate discontinuation of the oral contraceptives. Hepatic CH, focal nodular hyperplasia, hepatocellular adenoma, and hepatic peliosis have all been associated with the use of oral contraceptives and related to pregnancy [6–9]. Hepatic peliosis, which is sometimes confused with DHH, is a lesion characterized by sinusoid dilatation. On CT scans, it is enhanced centrifugally, sometimes centripetally. Imaging findings differ depending on the presence or absence of bleeding or thrombus in the sinusoids. In our patient, a contrast-enhanced ultrasound showed a vascular-like structure that was thought to be sinusoidal dilation, and hepatic peliosis was suspected. Hepatic peliosis has atypical findings and is difficult to diagnose with imaging only.

A search of the literature revealed two cases of DHH complications associated with the administration of estrogen medications [10]. Although no growth of the lesions was observed after the medications were discontinued in both of the reported cases, the lesion grew after the drug withdrawal in our patient. Therefore, we chose to resect the lesions.

Jhaveri et al. studied the association of hepatic hemangiomatosis with a giant CH. They reported no association between the size of CH and the presence and extent of hepatic hemangiomatosis, and that hemangiomatosis was not rare in the liver parenchyma adjacent to a giant CH [5]. DHH often exists in the same lobe of CH, but, in some cases, spreads to both lobes of the liver.

Seven surgical cases for DHH have been reported, including our case (Table 1). Six of these cases were symptomatic. Our patient was the only case with a history of oral contraceptive use. Five cases were associated with CH and one case involved both lobes of the liver. Three patients underwent hepatic lobectomy, one underwent an extended hepatectomy, and two underwent liver transplantation. Though the duration of follow-up in reported cases varies, the prognosis of DHH treated with resection is good with no recurrence in five cases and improvement in all symptomatic cases. In unresectable cases, anti-VFGF agents, radiation therapy, and arterial embolization have been reported to improve symptoms, but it is difficult to control the disease in a rapidly progressing case [16–19]. Although CH is a benign tumor, hemangiomatosis is severe as the CH grows, and KMS may also occur [18]. Extensive DHH is difficult to resect considering remnant liver volume and no residual lesions, so the timing of resection should be carefully considered.

**Table 1:** Summary of reported case of surgery treatment for DHH

Case	Author	Age	Sex	Symptoms	Oral contraceptive usage	Location	With cavernous hemangioma	Treatment	Clinical course
1	Lehmann et al. [11]	35	Female	Abdominal pain, night sweat, fever	No	Right lobe	Yes	Right lobectomy	Residual liver recurrence after 6 years of surgery
2	Moon et al. [12]	50	Female	Abdominal discomfort, indigestion	No	Right lobe	Yes	Right lobectomy	No recurrence for 9 month
3	Maeda et al. [13]	47	Female	Abdominal distention	No	Both lobe	No	Living donor liver transplantation	No recurrence for 2 years
4	Maeda et al. [13]	31	Female	Abdominal distention	No	Both lobe	No	Extended right lobectomy	No progression of remaining hemangiomatosis
5	Ohkura et al. [14]	35	Female	Abdominal pain	No	Right lobe	Yes	Right lobectomy	Discharged on PODS
6	Lee et al. [15]	50	Female	Dyspnea, Abdominal pain	No	Both lobe	Yes	Living donor liver transplantation	Discharged on POD11
7	Our case	29	Female	Asymptomatic	Yes	Left lobe	Yes	Left lobectomy	Discharged on POD12. No recurrence for 1 year

*POD* Postoperative day

Though our patient remained asymptomatic, her lesion was growing and we did not want to miss the optimal timeframe for resection. The scattered distribution of DHH can lead to challenging surgical boundaries during resection. We used intraoperative ultrasounds and surgical findings to determine the appropriate resection line for our patient.

As DHH is a rare condition, there are few reports on the prognosis for patients. The recurrence of lesions after resections has been reported [11]. Thus, proper care must be taken in the long-term management of these patients. Resections are challenging when the lesion has spread to both lobes, and surgeons should carefully consider operating approach before this occurs.

## Conclusion

We report a case of the successful resection of hepatic CH with DHH in a patient who had a history of oral contraceptive use. The resection of these lesions should be carefully timed by surgeons.

## Data Availability

Not applicable.

## Abbreviations

CH: Cavernous hemangioma
DHH: Diffuse hepatic hemangiomatosis
KMS: Kasabach–Merritt syndrome
CT: Computed tomography
MRI: Magnetic resonance imaging
Gd-EOB-DTPA: Gadolinium-ethoxybenzyl-diethylenetriamine pentaacetic acid
POD: Postoperative day
